# Research progress on the correlation between gut microbiota and preeclampsia: microbiome changes, mechanisms and treatments

**DOI:** 10.3389/fcimb.2023.1256940

**Published:** 2023-10-31

**Authors:** Yichi Zong, Xuguang Wang, Jun Wang

**Affiliations:** ^1^ Department of Obstetrics and Gynecology, Shengjing Affiliated Hospital of China Medical University, Shenyang, Liaoning, China; ^2^ Sun Yatsen University Cancer Center, Guangzhou, Guangdong, China

**Keywords:** gut microbiota, preeclampsia, microbiome-immune axis, microecological therapy, probiotics

## Abstract

Preeclampsia is a specific disease during pregnancy and is a significant factor in the increased mortality in perinatal women. Gut microbiota, an intricate and abundant microbial community in the digestive tract, is crucial for host metabolism, immunity, and nutrient absorption. The onset and progression of preeclampsia are closely correlated with the changes in maternal gut microbiota. Research purpose was to compile the existing bits of present scientific data and to close the gap in the knowledge of changes in gut microbiota in preeclampsia and their association with preeclampsia. We searched studies from two electronic databases (PubMed and Web of Science) included from 2014 to 2023. This review is divided into three parts. In the first part, the author elaborates longitudinal differences of maternal gut microbiota during different gestation periods. In the second part, we discuss that gut microbiota can lead to the occurrence of preeclampsia by systemic immune response, influencing the release of active peptides, short-chain fatty acids, trimethylamine-N-oxide (TMAO) and other metabolites, vascular factors and Microorganism-immune axis. In the third part, we proposed that a high-fiber diet combined with drugs and microecological regulators may be therapeutic in enhancing or preventing the emergence and evolution of preeclampsia, which needs further exploration. Although the pathogenesis of preeclampsia is still nebulous and there is no clear and valid clinical treatment, our study provides new ideas for the pathogenesis, prevention and treatment of preeclampsia.

## 1 Introduction

**Figure 1:**
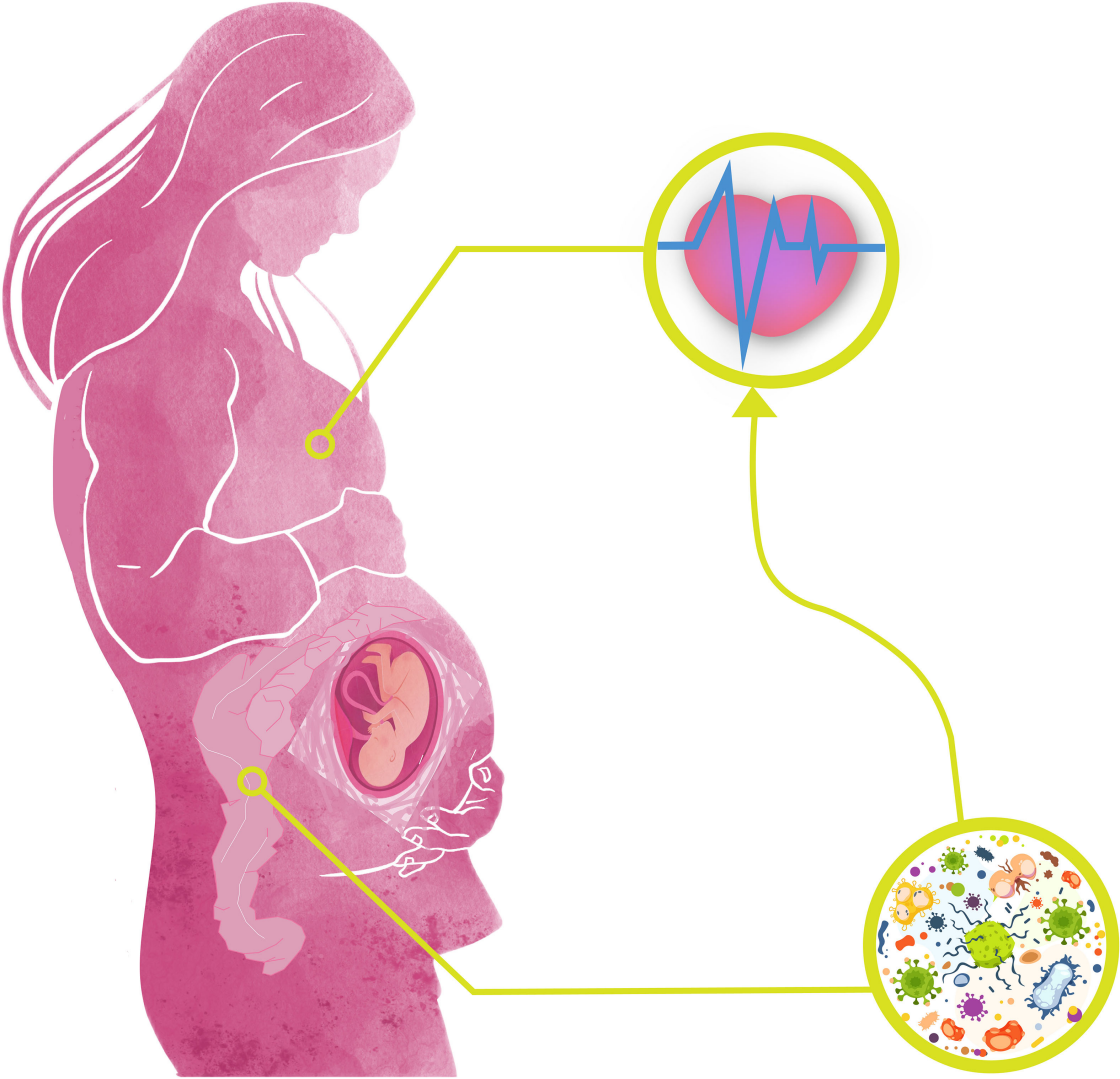
Gut microbiota and preeclampsia.

Preeclampsia is a specific disease during pregnancy and is a chief factor of increased mortality in perinatal women. It is defined by newly onset hypertension (systolic blood pressure ≥ 140 mmHg or diastolic blood pressure ≥ 90 mmHg) and significant proteinuria after 20 weeks of pregnancy. When the disease develops seriously, it might develop into eclampsia or be accompanied by multiple organ failure (Phipps et al., 2019). Compared with normal pregnant women, patients with preeclampsia are substantially more likely to develop cardiovascular disease in the future (Leslie and Briggs, 2016). In recent years, despite countless investigations and research conducted on preeclampsia, there are different opinions on its etiological hypothesis, including vascular endothelial damage, shallow placental implantation, genetic susceptibility, insulin resistance, oxidative stress, and nutritional deficiency (Udenze, 2016; Jabalie et al., 2019). At of now, the pathogenesis of preeclampsia remains not fully clear, and there is a lack of effective preventive measures. Symptomatic treatment is often taken after corresponding clinical symptoms appear in the middle or late pregnancy, resulting in poor prognosis for pregnant women and newborns (Brown et al., 2018). Therefore, an in-depth exploration of the pathogenesis of preeclampsia can present new potential targets for the early diagnosis and management of preeclampsia, which might is essential in preventing the occurrence of preeclampsia and improving the prognosis of patients with preeclampsia. There are many bacteria, viruses, and fungi in the human intestine. Among these symbiotic microbiota within the human body, the largest and richest microbiota is the intestinal microbiota (Pevsner-Fischer et al., 2017). Intestinal microbiota is a complex and abundant microbial community in the digestive tract, which is crucial for host metabolism, immunity, and nutrient absorption (Viennois and Chassaing, 2018). The number of gut microbiota cells (10^14^) is about 10 times that of human cells (10^13^), and the total mass is about 1.5kg. The total genome size of such a large number of bacteria is about 100 times that of human genome (Sommer and Backhed, 2013).There are four primary categories in the gut microbiota’s composition: *Firmicutes, Bacteroides, Actinomycetes*, and *Proteus.* Among them, the vast majority (90%) belong to *Firmicutes* and *Bacteroidetes*. The ratio of *Firmicutes* to *Bacteroidetes* (F/B) is an important parameter reflecting the disorder of gut microbiota. In addition, the richness, diversity, and evenness of gut microbiota are also crucial indicators reflecting the species diversity of gut microbiota. The equilibrium between physiology and immunological status is significantly maintained by the steady state of the gut microbiota (Collado et al., 2015). An irregularity in the composition of intestinal microbiota can cause metabolic abnormalities and systemic inflammation, which are responsible for the development of numerous illnesses. There is mounting evidence that intestinal microbiota and its related metabolites are essential to the incidence and development of cardiovascular diseases namely hypertension (Yang et al., 2015), atherosclerosis (Karlsson et al., 2012), and heart failure (**Figure 1**) (Yang et al., 2015; Tang et al., 2017).

While the correlation between gut microbiota and preeclampsia is still in early stages of development, a considerable amount of academic research has accumulated in this field, indicating that the relationship between the two is complex and multi-dimensional. A comprehensive review of the relevant literature will be especially helpful in synthesizing the key research insights and unveiling major research trends in this field. Hence, by reviewing relevant literature, this study intends to answer the following research questions: Changes of gut microbiota in pregnant women with preeclampsia; Mechanism of microbiota imbalance affecting preeclampsia; Methods and approaches for prevention and treatment of preeclampsia. It is a systematic review of the three focused questions.

## 2 Methods

We followed the guidelines of the Preferred Reporting Items for Systematic Reviews and Meta-Analyses (PRISMA) to conduct and report the present review (Liberati et al., 2009).

### 2.1 Information sources

We searched studies from two electronic databases (PubMed and Web of Science) included from 2014 to 2023.

### 2.2 Search strategy

We used various combinations of MeSH descriptors associated with the text words: “ Pre Eclampsia OR Preeclampsia OR Pregnancy Toxemias OR Pregnancy Toxemia OR Toxemia, Pregnancy “ AND “Gastrointestinal Microbiomes OR Microbiome, Gastrointestinal OR Gut Microbiome OR Gut Microbiomes OR Intestinal flora”.

### 2.3 Selection process

Articles were considered for inclusion based on the PICOS strategy, as follows: Population mainly refer to PE patients in early, middle, and late pregnancy. Interventions were changes in gut flora during early, middle, and late pregnancy. Comparator were women with normal pregnancy. Outcome comprised preeclampsia and its criteria reported by the authors; Study comprised observational studies: cross-sectional studies, case-control studies, cohort studies, written in English, without time restriction. We excluded any article not reporting original results (reviews, abstracts, editorials, comments).

### 2.4 Data extraction

The following data from studies included in the quantitative synthesis were collected and tabulated by two independent reviewers(Yichi Zong, Jun Wang using a standardized data extraction form: (i) first author name, (ii) publication year, (iii) sample size, (vii) number of cases, (ix) gestation periods, (x) gut microbiota increased and decreased (**Table 1**).

**Table 1:** Changes of intestinal flora in preeclampsia during different pregnancy periods.

Reference	Model	Stage	Increase	Decrease
Chen et al. (2020) (Chen et al., 2020)	NP(n=85) PE(n=67)	T3	—— Clostridium Dialister Fusobacterium Veillonella	—— Faecalibacterium Akkermansia Lachnospira
Wang et al. (2019) (Wang et al., 2019)	NP(n=48) PE(n=48)	T1, T2, T3	—— Bacteroidetes Proteobacteria Actinobacteria Bacteroidia Bacteroides coprocola Bacteroides fragilis Enterobacteriaceae Gammaproteobacteria Enterobacteriales	—— Firmicutes Faecalibacterium Clostridia Clostridiales Ruminococcus Rikenellaceae Alistipes Bacteroides stercoris
Lv et al. (2019) (Lv et al., 2019)	NP(n=72) PE(n=78)	T1, T2, T3	—— Blautia Ruminococcus Bilophila Fusobacterium Anaerococcus Oribacterium Parvimonas Abiotrophia	—— Faecalibacterium Gemmiger Akkermansia Dialister Methanobrevibacter Fusobacteria Tenericutes Verrucomicrobia
Liu et al. (2017) (Liu J. et al., 2017)	NP(n=74) PE (n=26)	T1, T2 T3	Verrucomicrobia Tenericutes	—— Verrucomicrobia
		T3	Cyanbacteria Bulleidia moorei Clostridium perfringens	Firmicutes Coprococcus catus
Zhao et al. (2023) (Zhao et al., 2023)	NP (n=45) PE (n=41)	T2, T3 T2, T3	—— Proteobacteria, Fusobacteria, class Erysipelotrichi, Bacilli, Fusobacteria, Gammaproteobacteria, order Erysipelotrichales, Enterobacteriales, Fusobacteriales, Lactobacillales, Family Erysipelotrichaceae, Enterobacteriaceae, Fusobacteriaceae, Genus Shigella, Fusobacterium, Streptococcus	—— Bacteroidetes, class Bacteroidia, order Bacteroidales, family Bacteroidaceae, Ruminococcaceae, genus Bacteroides, Ruminococcus, Oscillospira
Huang et al. (2021) (Huang et al., 2021)	NP(n=28) PE(n=26)	T3 T3	—— ——	Proteobacteria Tenericutes Lactobacillus Prevotella Porphyromonas Varibaculum Lactobacillus
Wang et al. (2020) (Wang et al., 2020)	NP(n=25) PE(n=25)	T3 T3	—— Bacteroidetes Proteobacteria Enterobacteriaceae class Bacteroidia order Bacteroidales	Proteobacteria Tenericutes Firmicutes class Clostridia, order Clostridiales, genus unidentified Lachnospiraceae
Chang et al. (2020) (Chang et al., 2020)	NP(n=36) PE(n=27)	T1, T2, T3 T1, T2, T3	—— Proteobacteria Gammaproteobacteria Enterobacteriales Enterobacteriaceae Veillonellaceae Escherichia_Shigella	—— Firmicutes Clostridia Clostridiales Bifidobacteriales Lachnospiraceae Ruminococcaceae Streptococcaceae Bifidobacteriaceae Blautia Streptococcus Eubacterium_rectale Eubacterium_hallii *Bifidobacterium*
Lv et al. (2022) (Lv et al., 2022)	NP(n=37)	T3	Blautia, Pauljensenia, Ruminococcus, Collinsella.	——
	PE(n=40)	T3	Bacteroides, Phocaeicola	——
Meijer et al. (2023) (Meijer et al., 2023)	NP(n=29) PE(n=25)	T3 T3	phylum Bacteroidetes	phylum Verrucomicrobia phylum Syntergistota genus Akkermansia
Miao et al. (2021) (Miao et al., 2021)	NP(n=8)	T3	——	——
	PE(n=12)	T3	genus Blautia Ruminococcus	phylum Actinobacteria family Bifidobacteriaceae genus *Bifidobacterium*

NP, Normal pregnant women; PE, Preeclampsia pregnant women preeclampsia pregnant women.

### 2.5 Study selection and characteristics

**Figure 2:**
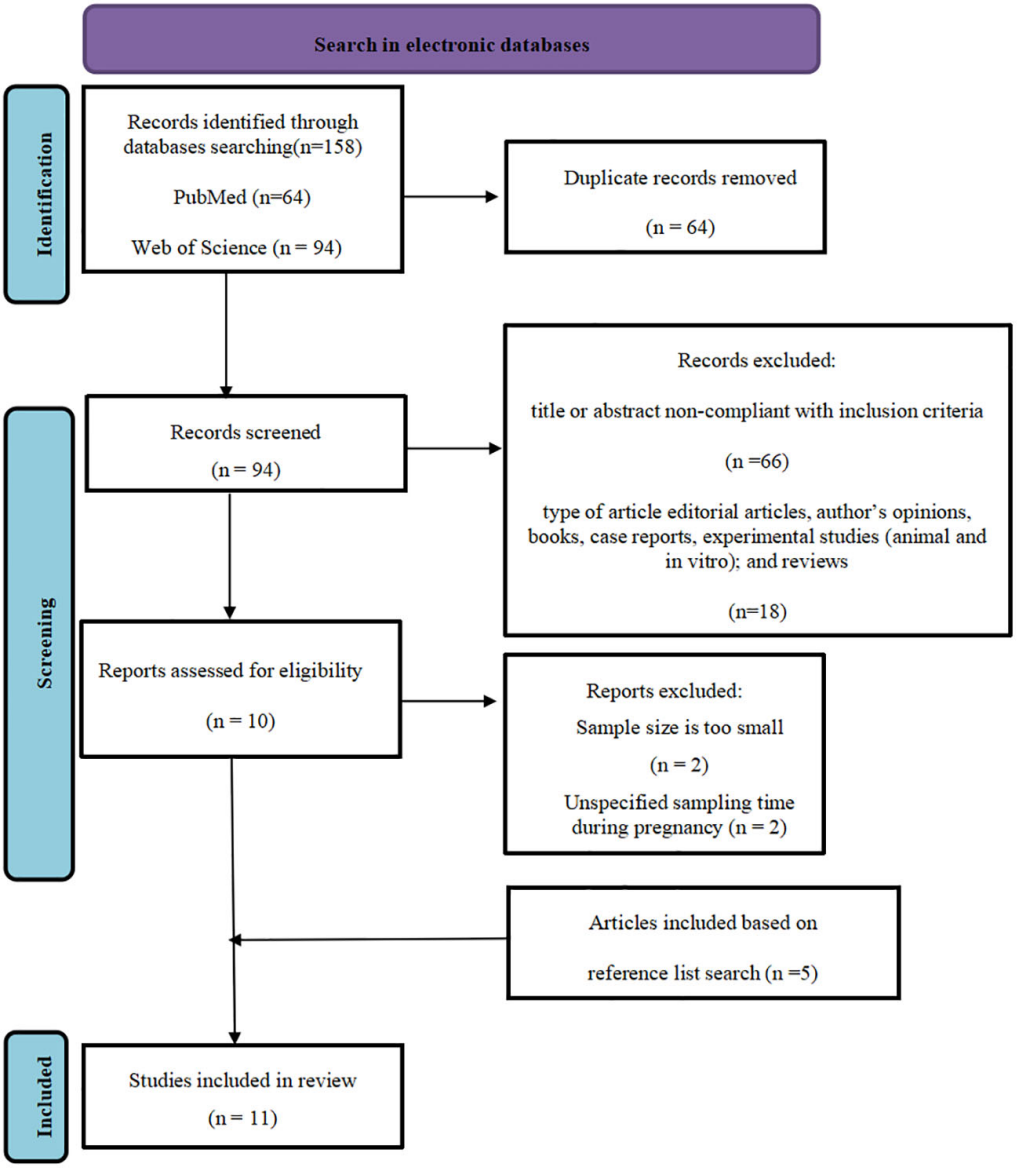
PRISMA flowchart of search strategy, screening, eligibility and inclusion criteria.

The literature search retrieved 158 potentially eligible studies. Of these, 11 met the inclusion criteria. All the included studies were from two electronic databases (PubMed and Web of Science) included from 2014 to 2023.The search was conducted in each database separately and then combined to pool together all the literature. The pooled search database was examined and the duplicates were removed and appropriate limits were applied (Duplicates were removed by using Endnote software version X9), comprising 64 articles. The main reason for exclusion were that the title or abstract out of scope and the type of article were not observational studies, comprising 84 articles. The samples of 2 articles were too small, and the sampling time during pregnancy in 2 articles was uncertain. 5 articles based on reference list search was included. The flow chart of the systematic review is available in (**Figure 2**) (PRISMA template).

## 3 Changes of maternal gut microbiota in healthy pregnant women and preeclampsia during different gestation periods

### 3.1 Changes of maternal gut microbiota during pregnancy

Koren et al. proposed that with the progress of pregnancy, the component part of gut microbiota changed greatly from early pregnancy to late pregnancy. The gut microbiota’s makeup in early pregnancy have something in common with that of non-pregnant women and men: *Bacteroides, Firmicutes, Actinomycetes*, and *Proteus* are four comparatively constant bacteria in gut microbiota during pregnancy. Most of the dominant bacteria are *Clostridium* of *Firmicutes* (Koren et al., 2012). At the species level, eight bacterial species were enriched in healthy controls: *Alistipes putredinis*, *Bacteroides vulgatus*, *Ruminococcus torques*, *Oscillibacter unclassified*, *Akkermansia muciniphila*, *Clostridium citroniae*, *Parasutterella excrementihominis* and *Burkholderiales bacterium_1_1_47* (Lin et al., 2022).

In the study of Liu et al., the content of *Verrucomicrobia* was higher in the first trimester of pregnancy (T1) and the second trimester of pregnancy (T2) but almost disappeared in the third trimester of pregnancy (T3), with the number of *Tenericutes* increased in T3 (Liu J. et al., 2017). While in Huang’s study, the relative abundance of *Proteobacteria* and *Tenericutes* declined considerably from T2 to T3 (Wang et al., 2020; Huang et al., 2021). Since Liu’s research is limited to pregnant women in southern China, the contradiction between the two results may be caused by regional or ethnic distinctions. In T3, the structure and composition of intestinal flora of pregnant women are similar to the disorder of disease-related flora and show strong mask of inflammation and energy loss. A typical sign is low diversity of bacteria. In addition, the abundance of *Faecalibacterium* with anti-inflammatory effect is reduced: the abundance of *Faecalibacterium* in T3 is substantially lower than that in T1, and as a butyrate producing bacterium, it has an important inhibitory effect on inflammatory response. Lastly, the abundance of *Actinobacteria* and *Proteobacteria* related to chronic low-grade inflammation increased: the proportion of *Proteus* and *Actinomycetes* increased (Koren et al., 2012). The serum progesterone increases gradually after pregnancy, and reaches a relatively higher level in T3. Nuriel et al. found that progesterone can affect intestinal flora by accelerating the growth of *Bifidobacterium* in T3. Therefore, the maternal gut microbiota in T3 can show an increase in the relative abundance of *Bifidobacterium* (Nuriel-Ohayon et al., 2019). Li et al. found that *Bifidobacterium* was associated with PE (Li et al., 2022).The metabolic gene of human milk oligosaccharide (HMO) expressed by *Bifidobacterium* activates the tryptophan metabolic pathway and increases the metabolite of indole-3-lactic acid, which promotes the anti-inflammatory cytokine interleukin-27 (IL-27) and interferon β (IFN-β), and induces T cells to secrete regulatory factor galectin-1, thereby maintaining the homeostasis of intestinal and peripheral immune system and inhibit excessive immune response (Henrick et al., 2021). Progesterone has a positive impact on the microbial composition during pregnancy, which can be beneficial for the mother’s health and also support the transfer of beneficial gut microbiota to the newborn (Nuriel-Ohayon et al., 2019).

### 3.2 Changes of maternal gut microbiota in preeclampsia

There is evidence that preeclampsia is related to changes of maternal gut microbiota. A study in *Gut* analyzed fecal samples from 67 pregnant women with preeclampsia and 85 normotensive pregnant women, and found reduced gut bacteria diversity among participants with the condition, with clear imbalances of healthy vs disease-associated strains. Women with preeclampsia had more opportunistic pathogens, particularly *Fusobacterium* and *Veillonella* for example, and fewer beneficial bacteria, including *Faecalibacterium* and *Akkermansia*. These imbalances correlated with blood pressure levels and markers of renal insufficiency, hinting at a possible link to preeclampsia, which is identified by hypertension and often signs of multiple organ damage (Chen et al., 2020). Wang et al. found that the abundances of *Bacteroidetes*, *Proteobacteria*, *Actinobacteria*, *Enterobacteriaceae* and *gamma-Proteobacteria* were higher, while the abundance of *Firmicutes*, *Faecalibacterium*, *Clostridia*, *Clostridiales* and *Ruminococcus* were lower (Wang et al., 2019). The study of Lv also proved that the presence of PE-enriched genera such as *Anaerococcus*, *Ruminococcus*, and *Oribacterium* showed a positive correlation with maternal blood pressure and liver enzyme levels (Lv et al., 2019). To explore a relationship between gut microbiota dysbiosis and disease progression, it is essential to conduct prospective studies that focus on longitudinal variations occurring during gestation in different trimesters. In the phylum level, the composition analysis showed that the most plentiful microbes in normal pregnant women (T1, T2, T3) and PE pregnant women are *Bacteroidetes* (47.6–52.4%), *Firmicutes* (35.9–41.4%), *Proteobacteria* (3.0–8.3%) and *Actinobacteria* (1.1–4.8%). Gut microbiota were analyzed by sequencing the V4 region of the 16S rDNA gene using Illuminal MiSeq. (Liu J. et al., 2017). There were significant differences in *Bacteroidetes*, *Proteobacteria*, and *Fusobacteria* between the two groups at the phylum level. Microbial α diversity was lower in PE patients than that in healthy controls. In addition, there was significant difference in microbial β diversity between the two groups (Zhao et al., 2023). The abundance of three bacteria in *Firmicutes*, including *Clostridiu perfringens, Bulleidia moorei*, and *Coprococcus catus*, varied significantly between T3 normal pregnant women and preeclampsia expectant women. In the preeclampsia group, the relative abundance of *Clostridium perfringens* and *Bulleidia moorei* was considerably higher, while the relative abundance of beneficial bacteria feline *Coprococcus catus* was considerably lower. The end result of these changes is that the abundance of *Firmicutes* decreased in pregnant women with PE. In the phylum level, the fifth most abundant group was *Cyanobacteria.* Women with PE showed higher level of *Cyanobacteria* (Huang et al., 2021). In Hang’s study, the gut microbiota altered significantly from T2 to T3. The gut microbiota of patients in T3 of PE was substantially distinct from that of normal pregnant women. The relative abundance of *Bacteroidetes* in T2 phase of PE pregnant women was lower than that in T3 phase. In T2 phase, there was no apparent variation in the relative abundance of microbiota between PE pregnant women and normal pregnant women. However, in T3 phase, the relative abundance of *Firmicutes* in PE pregnant women was considerably lower than that in normal pregnant women. The relative abundance of *Bacteroidetes, Proteobacteria*, and *Enterobacteriaceae* in PE pregnant women was considerably higher than that in normal pregnant women (Wang et al., 2020). During T3 in pregnant women with PE, there was an increase in the relative abundance of taxonomic groups including *Bacteroidetes*, *class Bacteroidia*, and *order Bacteroidales*. Conversely, there was a decrease in the abundance of taxonomic groups including the *phylum Firmicutes*, the *class Clostridia*, the o*rder Clostridiales*, and the *genus unidentified Lachnospiraceae* (Wang et al., 2020).

These changes were identified as taxonomic biomarkers of PE, suggesting a potential relationship between these microbial groups and the development of PE (Wang et al., 2020; Huang et al., 2021). Chang et al. found that healthy pregnant women (NP) and PE had significantly different abundances from phylum to genus levels. Specifically, the NP group had higher concentrations of *Eubacterium_rectale*, *Eubacterium_hallii*, *Firmicutes*, *Clostridia*, *Clostridiales*, *Bifidobacteriales*, *Lachnospiraceae*, *Ruminococcaceae*, *Streptococcaceae*, *Bifidobacteriaceae*, *Blautia*, *Streptococcus*, and *Bifidobacterium*, whereas *nterobacteriales*, *Enterobacteriaceae*, *Veillonellaceae*, *Proteobacteria*, *Gammaproteobacteria*, *E* and *Escherichia_Shigella* were more abundant in the PE group (Chang et al., 2020). Enriched *Bacteroidetes* and depleted *Verrucomicrobia* and *Syntergistota* at the phylum level and depleted *Akkermansia* at the genus level, suggesting that it played a role in the pathophysiology of PE during the T3 phase (Meijer et al., 2023). Miao et al. noted a decreased abundance of bacteria of the *phylum Actinobacteri*, decreased abundance of bacteria of the *family Bifidobacteriaceae*, increased abundance of bacteria of the *genus Blautia* and *Ruminococcu*, and decreased abundance of bacteria of the *genus Bifidobacteriu* in PE group during the T3 (**Table 1**) (Miao et al., 2021).

## 4 Roles of gut microbiota in the pathogenesis of preeclampsia

**Figure 3:**
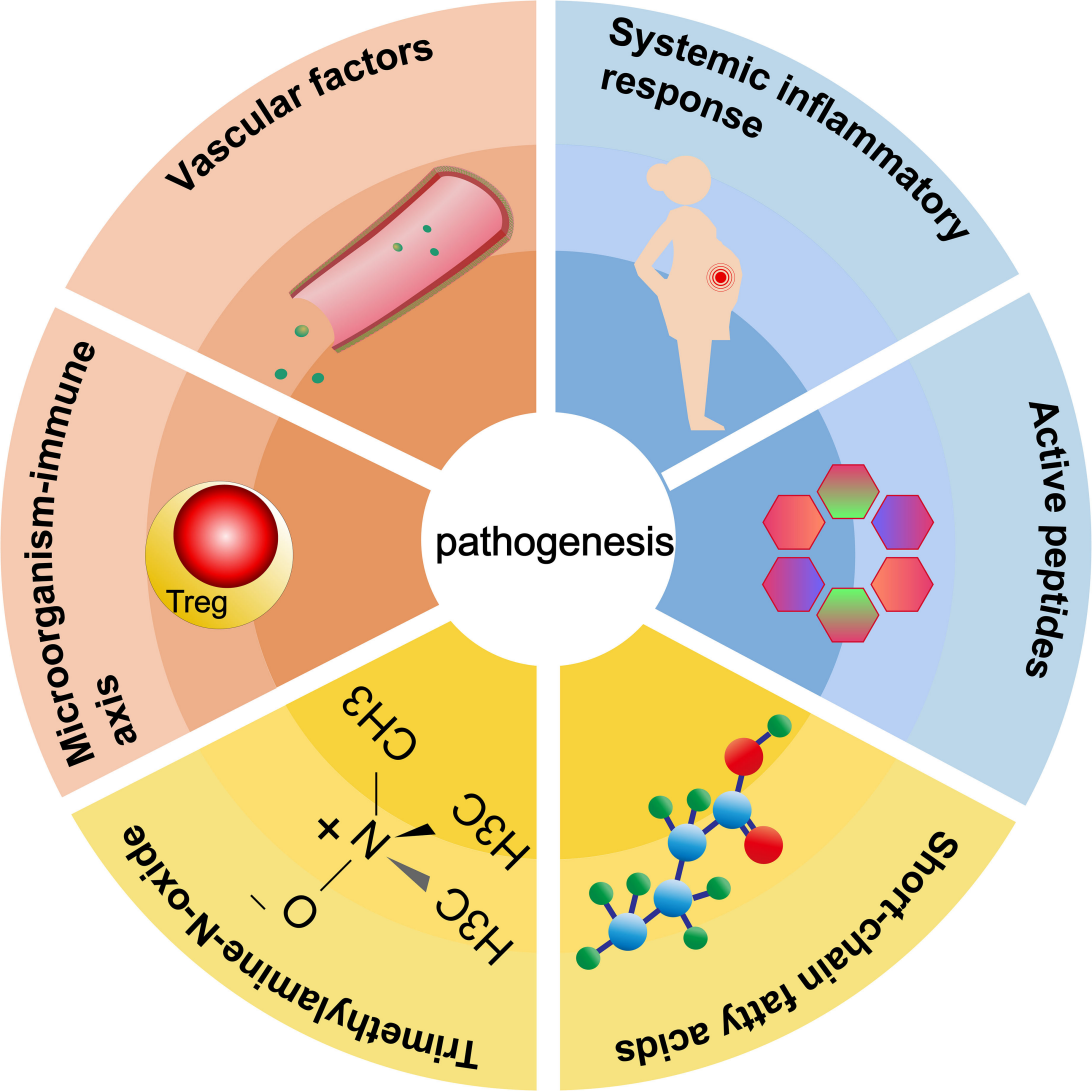
Six roles of gut microbiota in the pathogenesis of preeclampsia.

Pathogenesis of preeclampsia is related to vascular factors, imbalance of immune balance, systemic inflammatory response, regulation of active peptides, short-chain fatty acids, trimethylamine oxide, and other metabolites (**Figure 3**).

### 4.1 Gut microbiota and systemic inflammatory response

Blood pressure can be regulated by gut microbiota through influencing the host’s inflammatory response and modifying endothelial function (Gomez-Arango et al., 2016). A diversity of normal gut microbiota, such as *Clostridium* and *Bacteroides*, can regulate immunity through TGF-β signal transduction pathway. In mucosal tissues (such as intestinal or nasal mucosa), TGF-β can inhibit Th1 cell differentiation by suppressing the expression of IL-12 receptor β2, resulting in down-regulation of Th1 cell-mediated immune response, thus regulating Th1/Th2 balance (Kitani et al., 2000). *Bifidobacteria* and *Fusobacterium* are also the main variants of the PE microbiome. B. wadsworthia can facilitate a higher inflammatory response by generating hydrogen sulfide (da Silva et al., 2008), leading to intestinal barrier dysfunction and abnormal bile acid metabolism (Devkota et al., 2012; Natividad et al., 2018). Eghbal-Fard et al. (Eghbal-Fard et al., 2019) found that pregnant rats who received fecal bacteria transplantation of preeclampsia showed an imbalance of Treg/Th17, which aggravated placental inflammation, and the increase of inflammatory factors led to placental oxidative stress and vascular dysfunction, leading to maternal rejection of the fetus and the onset of hypertension.

Functional analysis using data gained from Kyoto Encyclopedia of Genes and Genomes (KEGG) showed significantly elevated lipopolysaccharide (LPS) biosynthetic pathways in the microbiota of patients with PE. The abundance of *Bacteroides* was substantially higher in the fecal microbiota of PE patients in T3. It is reported that members of *Bacteroides* are related to immune and metabolic processes (Lv et al., 2016). *Bacteroides*, a Gram-negative bacterium, is a major contributor to LPS biosynthesis. Consequently, high levels of *Bacteroides* may lead to an increase in inflammation during pregnancy. Gram-negative bacteria in the intestinal tract contain LPS, which is a potent activator of Toll-like receptor 4 (TLR4). The binding of LPS and TLR4 can stimulate a wide variety of cellular signal transduction pathways, thus leading to inflammatory response and cytokine expression and secretion, leading to the pathogenesis of preeclampsia. In PE patients, pro-inflammatory cytokines TNF-α and IL-6 in circulating and placental trophoblast cells increased, while anti-inflammatory cytokines IL-10 and IL-4 decreased. Many animal models use LPS to induce PE-like conditions in experiments (Cotechini et al., 2014). According to Liu Y. et al. (Liu Y. et al., 2017), injection of LPS into rodents may cause PE-like symptoms in the whole body and local placenta. LPS injection can successfully result in inadequate remodeling of placental spiral arteries as well as systemic and local inflammation. Meanwhile, a study found elevated plasma and fecal LPS levels in PE patients, which was in accordance with Zaman’s finding that serum LPS levels were higher in the PE group than in the normal pregnancy group (Wang et al., 2019). Metabolites of gut microbiota, such as short-chain fatty acids, especially propionate and butyrate, can prevent the expression of cytokines including IL-6, IL-12, and p40 in human mature dendritic cells by inhibiting LPS (Thursby and Juge, 2017).

The targeted microbial composition of fecal SIgA is strongly associated with the inflammatory status of pregnant women in the third trimester of pregnancy. SIgA targeted microbiota can distinguish late pregnant women into groups with different population characteristics. Previous research have demonstrated that SIgA can play a role in the inflammatory response, exerting a defensive effect by binding to fragment crystallizable (Fc) receptors like Human Receptor I for the Fc region of immunoglobulin A (FcARI) (Cui et al., 2020). FcaRI is expressed by neutrophils, eosinophils, monocytes, and macrophages throughout the intestine during inflammation, but not during homeostasis (Bakema and van Egmond, 2011). Thus, Fc receptors and complement have useful functions in the inflammatory state of the body, but it is unclear what part they play in normal intestinal microflora. In Cui’s study, gestational diabetes mellitus (GDM) and pregnancy induced hypertension (PIH) groups had high inflammatory markers (fecal calprotectin (FCALP) and LPS), demonstrating that there was a substantial relationship between the inflammatory response in pregnant women and the SIgA targeting microbiota (Cui et al., 2020).

### 4.2 Blood pressure is affected by released active peptides, short-chain fatty acids, trimethylamine-N-oxide and other metabolites

#### 4.2.1 The mechanism of released regulatory active peptides on blood pressure

*Bifidobacterium* is involved in the regulation and release of peptide fragments with antihypertensive activity. Intestinal microflora releases antihypertensive peptides from food proteins such as milk proteins through the hydrolysis of extracellular proteases and peptidases (carboxypeptidase, aminopeptidase) (Turpeinen et al., 2012; Chen et al., 2014; Ahren et al., 2015). Among them, *Bifidobacterium* can synthesize angiotensin converting enzyme inhibitory peptides, opioid active peptides and other substances with antihypertensive activity, competitively inhibit the activity of angiotensin converting enzyme, block the conversion of angiotensin I (Ang I) to angiotensin II (Ang II), and act on the renin-angiotensin system, thus reducing blood pressure (Hashimoto et al., 2012).

#### 4.2.2 Mechanism of trimethylamine oxide on blood pressure

Trimethylamine-N-oxide (TMAO) is one of the most significant metabolites generated by intestinal microflora. At the family level, *Enterobacteriaceae* contributed most to the transformation of TMAO to TMA (Hoyles et al., 2018), whereas *Deferribacteraceae* was engaged in the creation of TMA (Koeth et al., 2013). Intestinal bacteria metabolize dietary ingredients like choline and carnitine to TMA, and then oxidize TMA to TMAO through flavin-containing monooxygenase (FMO)(Velasquez et al., 2016). TMAO may participate in the pathogenesis of hypertension induced by Ang II (Ufnal et al., 2014). By encouraging the activation of NF-kB, TMAO increases the expression of inflammatory markers. In human umbilical vein endothelial cells, Sun et al. (Sun et al., 2016) demonstrated that TMAO can greatly increase the release of inflammatory cytokines including IL-1 and IL-18 while suppressing the secretion of NO. TMAO produced by dietary lecithin metabolism can predict the risk of cardiovascular disease, but its metabolism depends on gut microbiota, which can promote the up-regulation of macrophage scavenger receptors related to atherosclerosis, accelerate atherosclerosis and affect the pathological process of hypertension. At the same time, there is evidence that gut microbiota metabolize choline and phosphatidylcholine to produce TMAO, which can promote the formation of atherosclerotic plaque (Wang et al., 2011).

#### 4.2.3 Effect mechanism of short-chain fatty acids on blood pressure

**Figure 4:**
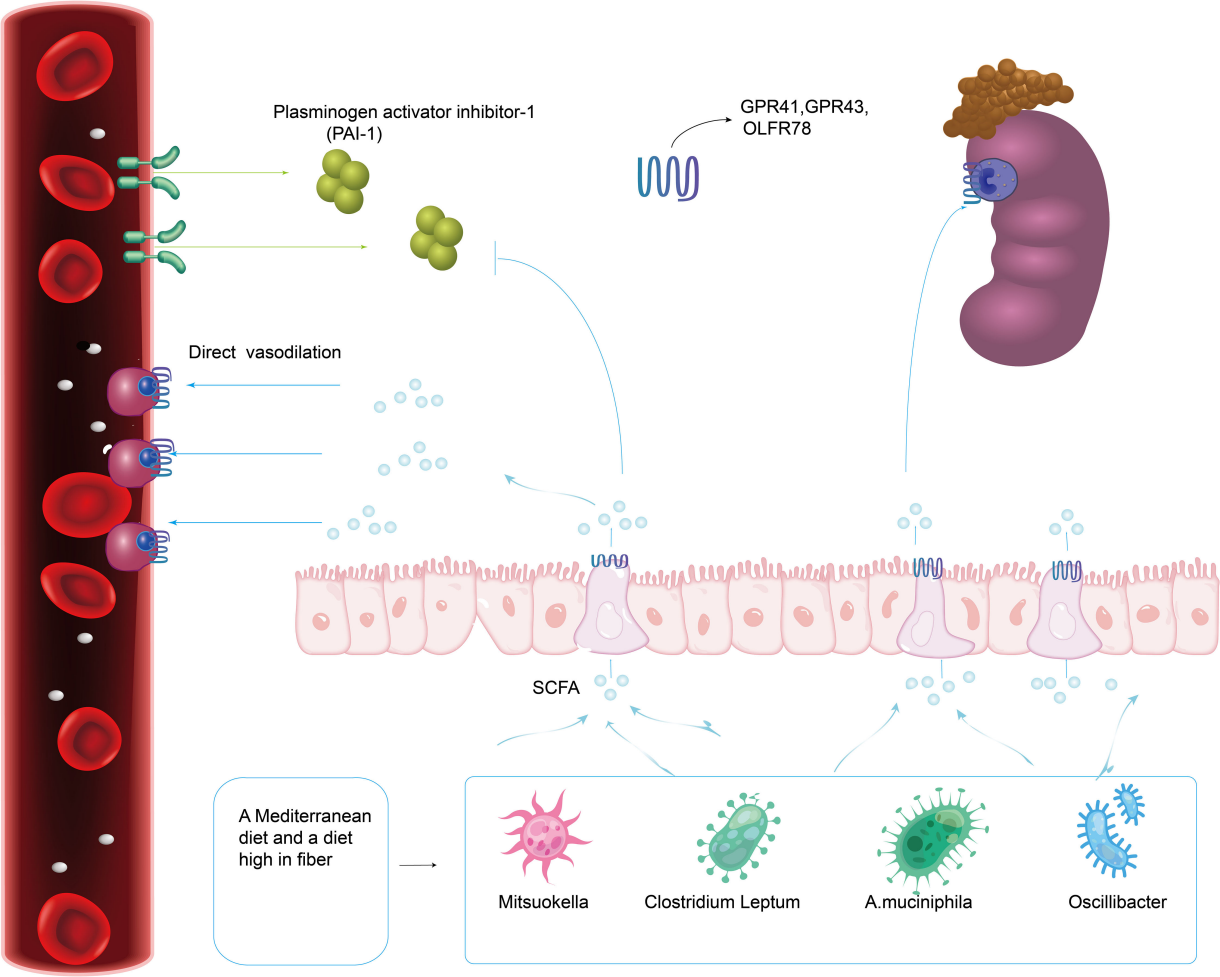
A Mediterranean diet as well as a diet high in fiber promotes the production of gut microbiota related to SCFA. Intestinal Microflora (Mitsuokella, Clostridium Leptum, A.muciniphila and Oscillibacter) can dilate blood vessels directly through SCFA receptors (GPR41, GPR43 and Olfr78) expressed in the gastrointestinal tract, smooth muscle cells of major blood vessels and kidney.

Short-chain fatty acids (SCFA), mainly acetate, butyrate and propionate, are the main metabolites of intestinal microbiota and are mainly created by bacterial fermentation of dietary fiber. Research have revealed significant compositional alterations and symbiosis of gut microbiota in preeclampsia patients, especially SCFA-producing bacteria such as *Mitsuokella*, *Clostridium Leptum*, *A.muciniphila* and *Oscillibacter*, which decreased significantly in abundance (Gomez-Arango et al., 2016; Liu J. et al., 2017; Lv et al., 2019). SCFA produced by intestinal microflora may regulate blood pressure and vasodilation mainly through G protein coupled receptor (GPCR), thus causing hypotension (Miyamoto et al., 2016; Poll et al., 2021). These effects are mediated through SCFA receptors, such as GPR41, GPR43, and olfactory receptor 78 (Olfr78), which are expressed in the gastrointestinal tract, smooth muscle cells of major blood vessels, and kidney (**Figure 4**) (Poll et al., 2021). Propionate’s binding to Olfr78 and Gpr41 regulates blood pressure by causing the release of renin, which is regulated through the creation of cyclic adenylate (cAMP) (Poll et al., 2021). The latest scientific research also shows that short-chain fatty acids can considerably decrease blood pressure in Ang II-induced hypertensive mice by regulating Gpr43 and Gpr109 (Kaye et al., 2020). Short-chain fatty acids can also activate intestinal epithelial cells to create chemokines and cytokines, mainly by acting on Gpr41 and Gpr43, and then activating extracellular signal-regulated kinases 1/2(ERK1/2)and p38 mitogen-activated protein kinase signaling pathways in epithelial cells, thus inducing the production of chemokines and cytokines in the process of immune response (Kim et al., 2013). According to Junki Miyamoto’s study, SCFA is concentration-dependent on the regulatory mechanism of blood pressure. 0.1~0.9 mmol/L short-chain fatty acids in plasma would stimulate Gpr41 to reduce blood pressure, and when the concentration of short-chain fatty acids is over 0.9 mmol/L, it would activate Olfr78 receptor, antagonize Gpr41, and ensure the dynamic balance of blood pressure (Miyamoto et al., 2016). Olfr78 receptors are expressed in vascular resistance beds and renal afferent arteries in many tissues. Animal experiments showed that plasma renin levels and baseline blood pressure levels were lower in Olfr78 knockout mice (Pluznick et al., 2013). Because the receptor can only be stimulated by acetic acid and propionic acid, It is hypothesized that the metabolites of gut microbiota may regulate blood pressure by downregulating Olfr78 (Natarajan and Pluznick, 2016). Gpr41 receptors are mainly expressed in smooth muscle cells of large vessels and kidney, and short-chain fatty acids are involved in renin secretion. Blood pressure in Gpr41 knockout mice increased by (4.5 ± 2.4) mmHg (1mmHg=0.133kPa) after exogenous propionate injection in Pluznick’s study. Their results showed that the main role of Gpr41 is to lower blood pressure (Pluznick, 2017). It is speculated that gut microbiota may reduce blood pressure by up-regulating the level of Gpr41.

### 4.3 Intestinal microflora and vascular factors

New evidence suggests that improper activation of the renin-angiotensin system (RAS) and alterations in intestinal microflora are related to the pathogenesis of hypertension (Pluznick et al., 2013; Yang et al., 2015; Al Khodor et al., 2017; Pluznick, 2017). RAAS exists in the intestine and plays a certain role (Richards et al., 2017).

**Figure 5:**
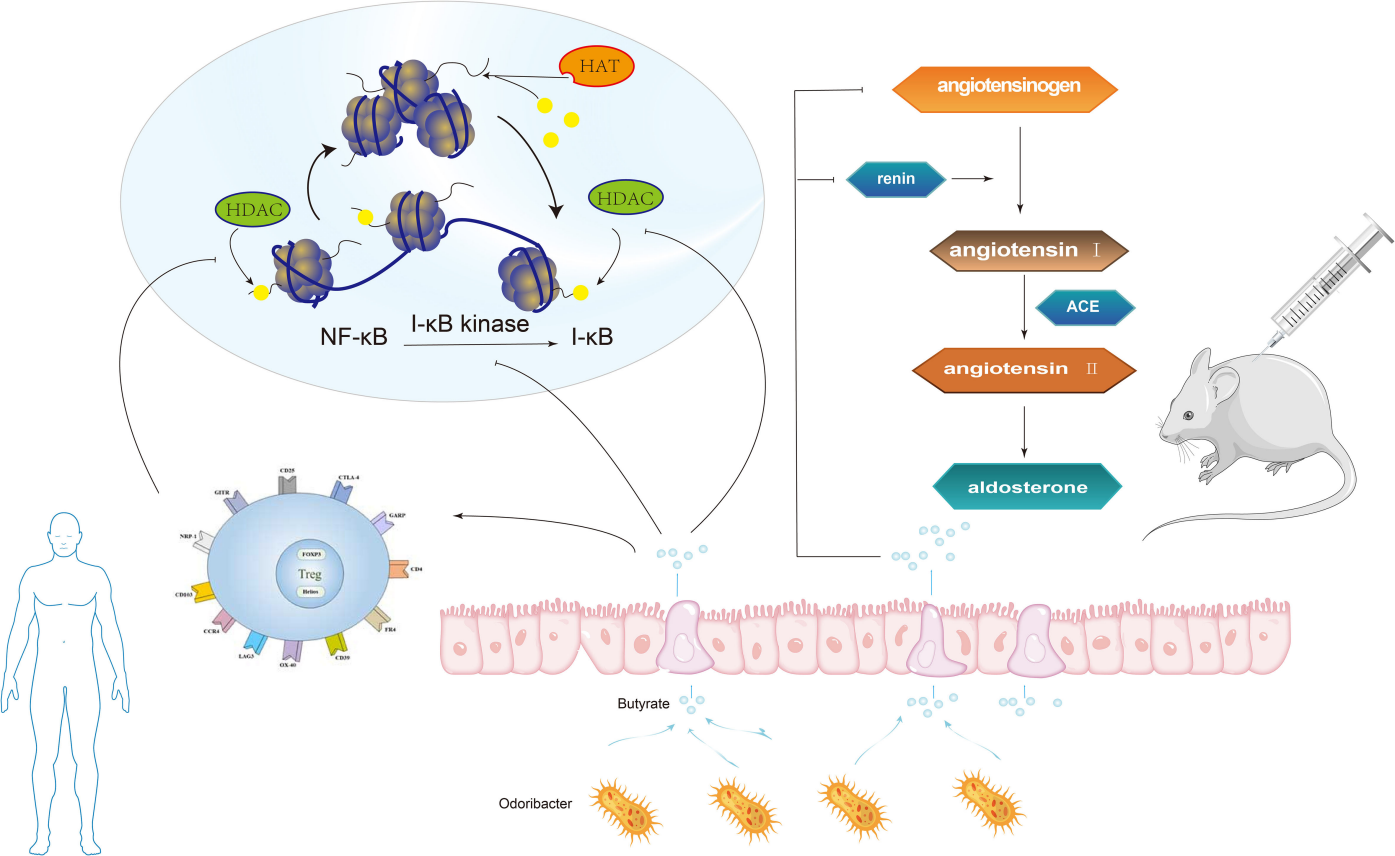
The butyrate produced by Odoribacter can inhibit NF-κB activation and histone acetylation (HDAC) in macrophages, in the human body. Intramedullary injection of butyrate can significantly reduce angiotensin II-induced hypertension in hypertensive rats by inhibiting the release of renin and angiotensinogen.

It has been reported that some intestinal symbiotic bacteria (eg. Cryptome) produce angiotensin-converting enzyme inhibitors, renin inhibitors, and antioxidant molecules during mucin digestion; and steroids that bind to bile salts in enterohepatic circulation can synthesize aldosterone, resulting in systemic vasoconstriction and impaired vascular endothelial function, leading to the pathogenesis of preeclampsia (Richards et al., 2017). Intramedullary injection of butyrate can dramatically lower Ang II-induced hypertension in hypertensive rats by inhibiting the release of renin and angiotensinogen (**Figure 5**) (Wang et al., 2017). SCFAs can regulate blood pressure by promoting vasodilation in rodents and humans (Mortensen et al., 1990; Nutting et al., 1991). Intestinal microflora can dilate blood vessels directly through SCFAs or affect blood pressure through plasminogen activator inhibitor-1 (PAI-1) (**Figure 4**).

Studies have found that PAI-1 levels in the placenta and circulation are abnormally increased in patients with gestational hypertension and high levels of PAI-1 in early pregnancy are linked to a high risk of hypertensive disorder in late pregnancy (Gomez-Arango et al., 2016). In a meta-analysis, the polymorphism of PAI-1 gene is linked to a higher risk of PE (Boe et al., 2013). In 2016, Gomez-Arango et al. (Gomez-Arango et al., 2016) reported that the abundance of *Odoribacter* in gut microbiota was negatively correlated with PAI-1. *Odoribacter* is a Gram-negative bacilli that can synthesize succinate, propionate, acetate, butyrate, isobutyrate, and isovalerate by fermenting carbohydrates *in vitro*. The butyrate produced by *Odoribacter* (**Figure 5**) can block the formation of PAI-1, thus mitigating the vasoconstriction and endothelial dysfunction triggered by endothelial nitric oxide synthase (eNOS) suppression. Therefore, lowering PAI-1 levels through intestinal microbiome metabolites may reduce the incidence of preeclampsia.

### 4.4 Microbiome-immune axis participates in blood pressure regulation

Studies using animal models of immune intervention, such as Toll-like receptor (TLR) deficient mice, suggest that TLR activation increases arterial pressure and causes vascular dysfunction, which also exists in spontaneously hypertensive rats (SHR) (Bomfim et al., 2012; Bomfim et al., 2015; McCarthy et al., 2015). Elevated blood pressure and vascular dysfunction are related to low-grade inflammation and gut microbiota imbalance, which reveals that there may be a microbial-immune axis involved in blood pressure regulation. Research have revealed that the composition of the immune system can be affected by the gut microbiota both directly and indirectly. First of all, the imbalance of bacteria, intestinal epithelial cells, and immune cells in the intestinal mucosa might lead to immune abnormalities mainly inhibited by Treg cells, which are a variety of CD4+T cell groups produced in the thymus and peripheral immune system (Tauschmann et al., 2013). Despite being spread throughout several phyla, the majority of the bacterial genera that produce propionate are rather small (Reichardt et al., 2014). Key organisms for making propionate and decomposing mucin have been found to include species like *Akkermansia Municiphilla* (Derrien et al., 2004). A surprisingly small number of organisms, dominated by *Faecalibacterium Prausnitzii*, *Eubacterium Rectale*, *Eubacterium hallii*, and *Ruminococcus bromii*, appear to produce the majority of the butyrate (Louis et al., 2010). Some studies have revealed that butyrate, a metabolite of gut microbiota, can inhibit NF-κB activation and histone deacetylase (HDAC) in macrophages (**Figure 5**). NF-κB is a eukaryotic transcription factor that controls many cellular responses, including immune and inflammatory responses. HDAC can inhibit the production of many pro-inflammatory cytokines in antigen-presenting cells (APC), promote the transformation of M1 macrophages into M2 macrophages, reduce TLR signals, disrupt antigen presentation, lessen the expression of major histocompatibility complex (MHC) class II and costimulatory molecules, reduce the direct inhibition of inflammation on Treg cells and APC stimulation, and prevent the inhibitory resistance of effector T cells (Teff) to Treg cells (Akimova et al., 2012; Morrison and Preston, 2016). The study also found that propionate and butyrate can play a potential role at the whole animal level by increasing the production and differentiation of Treg cells and inhibiting HDAC function (**Figure 5**) (Akimova et al., 2012). Secondly, during pregnancy, intestinal bacteria may translocate to placental tissue through dendritic cells in the intestinal tract (Abrahamsson et al., 2015), while butyrate can weaken gut microbiota translocation and increase intestinal barrier function by influencing tight junction assembly and mucin synthesis (Morrison and Preston, 2016; Thursby and Juge, 2017). Thirdly, Butyric acid can inhibit the activity of HDAC3, thus inhibit the transformation of M1 macrophages into M2 macrophages (the polarization process of macrophages). M2 macrophages can secrete matrix metalloproteinases (MMP), resulting in reduced ECM remodeling and shallow trophoblast invasion and poor spiral arteries remodeling to cause Preeclampsia. Therefore, gut microbiota can affect the incidence of preeclampsia by changing the polarity of macrophages and regulating the expression of MMPs.

## 5 Gut microbiota and the treatment of preeclampsia

### 5.1 Effect of diet on gut microbiota in patients with preeclampsia

A Mediterranean diet and a high-fiber diet that promotes SCFA (short-chain fatty acids) production are related with a reduction in occurrence of preeclampsia (**Figure 4**) (Qiu et al., 2008; Chen X. et al., 2016). In addition, supplementing with high fiber or taking acetate directly lowered systolic and diastolic blood pressure in a murine model, a finding highly relevant to preeclampsia (Marques et al., 2017). A high-fiber diet increases the number of bacteria known to have anti-inflammatory properties in women with gestational hypertension in the future, including Butyrate and Veronella. Marques et al. (Marques et al., 2017) gave a high-fiber diet and acetate diet respectively to Deoxycorticosterone hypertensive model mice. The results revealed that the high-fiber diet could reduce blood pressure by elevating the level of intestinal Bacteroides and acetate concentration. There is mounting evidence that diets high in fermentable fiber are beneficial to human gut microbiota and may raise the concentrations of SCFAs present in stool (Tan et al., 2014). Typically at the molar ratios of 60:25:15, acetate, propionate, and butyrate make up about 80% of the SCFA concentration in the gut lumen (Wong et al., 2006; Tazoe et al., 2008). In a study on whether gut microbiota changes in late-onset (>34 weeks gestation) preeclampsia (Altemani et al., 2021), serum butyrate levels, and acetate and propionate levels decreased significantly in developed late-onset (>34 weeks gestation) preeclampsia (DPE) compared with pregnant controls. This study revealed that the fecal SCFAs of the pregnant control group increased and the content of butyrate in PE decreased significantly (Chang et al., 2020). Developing PE was adversely correlated with lower serum acetate levels, according to a study that investigated maternal SCFA serum levels at 28 gestational weeks in 31 preeclampsia cases and 293 healthy pregnant women (Hu et al., 2019). In another study, hypertension caused by maternal intake of high-fructose (HF) was also associated with increased levels of isobutyric acid, isovaleric acid, and valeric acid in feces. In view of the vasodilating effect of acetic acid, acetate supplementation can prevent hypertension caused by maternal HF consumption (Hsu et al., 2019a). To determine whether butyrate supplementation may help reduce the incidence of preeclampsia, more research is necessary. The Mother and Child Cohort Study involving nearly 34000 pairs of mothers and infants in Norway between 2002 and 2008 showed that the incidence of severe preeclampsia in women who consumed high levels of fermented dairy products during pregnancy could be reduced by up to 40 percent. This effect is dose-dependent and less responsive to the intake of lower doses of fermented dairy products (Brantsaeter et al., 2011).

### 5.2 Effect of drugs on gut microbiota of preeclampsia

Resveratrol, a phytochemical, has demonstrated antioxidant properties and potential benefits in treating hypertension. Asymmetric dimethylarginine (ADMA)-related nitric oxide deficiency and gut microbiota-derived metabolite trimethylamine-N-oxide (TMAO) have been associated to hypertension. Resveratrol treatment rescued hypertension caused by the combination of ADMA and TMAO exposure. This was followed by changes in the gut microbiota’s composition and increased fecal butyrate levels (Hsu et al., 2021b).

Minocycline can rebalance the intestinal microflora of hypertension by reducing the ratio of thick-walled bacteria to *Bacteroides*, and the increase of blood pressure is also improved (Yang et al., 2015). However, a study validates the hypothesis that remodeling of intestinal microbiota at early crucial developmental stages impacts blood pressure in later years, and shows that taking minocycline during pregnancy and lactation period enhances blood pressure in offspring, which is consistent with decreases in acetate and butyrate (Hsu et al., 2021a). But the study has some limitations. The mechanisms mentioned in the study may not be fully capture the programming effects of minocycline therapy and HF intake, either separately or combined, on hypertension of developmental programming. Before this is applied in a clinical setting, further studies are needed in humans and other animal models of developmental programming.

A previous research investigated the interaction between amlodipine and combined antibiotics (ampicillin), and found that gut microbiota is involved in the metabolism of amlodipine. The intake of antibiotics may improve the bioavailability of amlodipine by inhibiting the metabolic activity of intestinal microorganisms (Yoo et al., 2016).

Intestinal microflora-dependent metabolites, especially trimethylamine (TMA), are associated with hypertension. In animal models, maternal exposure to 2,3,7,8-tetrachlorodibenzo-p-dioxin (TCDD) or eating high-fructose diet (HFR) foods can cause adult offspring to develop hypertension. In Andrea C. Aragon’s study, by making the pregnant dam dosed with TCDD, they found that prenatal TCDD exposure makes children more susceptible to Ang II-induced hypertension (Aragon et al., 2008). Some data have shown that TCDD aggravates the HFR-induced increase of blood pressure in adult male offspring, and this effect of TCDD can be prevented by maternal administration of 3, 3-maternal dimethyl-1-butanol (DMB), which is an inhibitor of TMA formation. The advantageous effect of DMB is linked to the change of intestinal microbiota, the increase of bioavailability of nitric oxide (NO), the balance of renin-angiotensin system, and the antagonism of aromatic hydrocarbon receptor (AHR) signal transduction (Hsu et al., 2020). The results of Tain et al. show that the protection of HFD-induced hypertension by ACE and DMB therapy is related to remodeling microflora composition and increasing microbial diversity (Tan et al., 2014). In the genus level, TMA and TMAO levels are favorably connected with *Prevotellaceae*, whereas they are negatively associated with *Akkermansia* (Chen et al., 2016). One study revealed that DMB lowered plasma levels of TMA and TMAO, enhanced the abundance of the species *Akkermansia*, and decreased the number of *Prevotellaceae*. It is possible that variations in a particular microbial community’s abundance influenced the balance of the TMA-TMAO pathway (Hsu et al., 2019a).

### 5.3 Microecological therapy of gut microbiota in preeclampsia

For many years, the treatment of preeclampsia has been restricted to symptomatic treatment or termination of pregnancy. Recent studies (Brantsaeter et al., 2011; Nordqvist et al., 2018; Sun et al., 2020) have found that supplementation of probiotics to regulate gut microbiota disorders may contribute in some ways to the prevention of preeclampsia. Probiotics are living microorganisms that, when taken in adequate amounts, may have positive effects on the host’s health. Probiotics are composed of single or multiple living bacterial species, such as *Lactobacillus* and *Bifidobacterium*, which can change the intestinal microflora during ingestion (Delzenne et al., 2011).

A HF diet during pregnancy has been found to adversely affect pregnancy and postpartum life (Regnault et al., 2013; Saad et al., 2016). Epidemiology has shown that excessive fructose intake is related to many common diseases, including hypertension (Johnson et al., 2007). An earlier study showed that the HF diet during pregnancy and lactation can lead to high blood pressure in adult offspring (Tain et al., 2015). Some reports suggest that the intake of 10-60% of high-fructose diets by pregnant women can lead to programmed hypertension in their adult offspring (Tain et al., 2015; Saad et al., 2016; Tain et al., 2016). Although a meta-analysis of 702 individuals showed that probiotic fermented milk had a hypotensive effect on adults with prehypertension or hypertension (Dong et al., 2013), the role of probiotics in reprogramming the way hypertension develops was not widely known. Hsu et al. (Hsu et al., 2018) presented evidence in their study that early probiotic treatment with *Lactobacillus casei* can inhibit programmed hypertension in adult offspring generated by maternal HF. Akin to probiotic therapy, studies have also shown that maternal prebiotic inulin treatment can prevent developmental hypertension caused by maternal HF intake. In addition, probiotics or prebiotics significantly increased the abundance of *Akkermansiamuciniphila*, a beneficial intestinal propionate-producing microorganism (Cani and de Vos, 2017). At the same time, probiotic or probiotic therapy can protect programmed hypertension caused by maternal HF diet associated with decreased abundance of *Bacteroides* acidis (Hsu et al., 2018). Data from a study suggest that altering gut microbiota through maternal prebiotic or probiotic treatment might assist in resolving hypertension caused by early overnutrition due to the HF diet (Hsu et al., 2019b). At the phylum level, Hsu et al. found that prebiotic or probiotic treatment inhibited HF-induced hypertension, and this effect was related to a decrease in *Firmicutes* abundance with a corresponding rise in *Bacteroidetes* and *Verrucomicrobia*. Meanwhile, prebiotic or probiotic treatment prevents against adult offspring’s high blood pressure caused by a rise in the abundance of *phylum Verrucomicrobia* and the *genera Lactobacillus* and *Akkermansia* (Hsu et al., 2019b). Probiotics such as *Bifidobacterium* and *Lactobacillus* can limit angiotensin converting enzyme activity and effectively lower blood pressure through proteolysis and fermentation. Probiotic intervention can reduce *Enterococci* and other pathogenic bacteria, improve intestinal barrier function, and regulate the level of Th1/Th2, thus reducing inflammatory reaction (Sun et al., 2020). Two studies of Norwegian in primiparae found that the incidence of preeclampsia decreased significantly in mothers with long-term intake of dairy products containing *Lactobacillus*, but had no protective effect on preterm delivery caused by severe preeclampsia (Brantsaeter et al., 2011). Intake dairy products containing *Lactobacillus* in the third trimester can considerably reduce the risk of preeclampsia (Nordqvist et al., 2018). Sun et al. (Sun et al., 2020) found that the colonization ability of intestinal microflora in preeclampsia rats treated with probiotics increased significantly; the level of endothelin-1 decreased and the level of nitric oxide increased, which reduced vascular endothelial cell injury, vascular permeability and inflammatory reaction to a certain extent, and helped to regulate the systolic blood pressure and diastolic blood pressure in the normal range.

Associations between clinical features and gut microbiota features offer potential evidence for future microbiota-targeted treatment, for instance, a decrease in the relative abundance of systolic blood pressure (SBP) and/or diastolic blood pressure (DBP) positively associated bacteria or treating potential probiotics (microbe inversely related to SBP and DBP) and/or their corresponding prebiotics. In the long term, probiotic supplements and special diets targeted by intestinal microflora might play a therapeutic role in treating or preventing preeclampsia, which is worth exploring.

## 6 Results

This paper reviews the changes of maternal gut microbiota in the first, second, and third trimester of women with preeclampsia:a time axis comparison. Pathogenesis of preeclampsia is related to vascular factors, imbalance of immune balance, systemic inflammatory response, regulation of active peptides, short-chain fatty acids, trimethylamine oxide, and other metabolites. The imbalance of gut microbiota can be improved by dietary adjustment and supplement of microecological regulators.

## 7 Conclusion

In conclusion, many studies have shown that there is a close relationship between preeclampsia and gut microbiota, and the imbalance of flora can impact the pathogenesis of preeclampsia by destroying immune tolerance, and inducing inflammatory reactions and metabolic disorders. At the same time, the maternal gut microbiota may influence the composition of the offspring’s gut microbiota. Through the detection of some metabolites produced by gut microbiota in blood, it may become an early warning signal of PE. Microecological agents have achieved certain expected results in improving inflammatory microenvironment and preventing PE. However, its specific mechanism and safety need to be further studied. A deeper comprehension of the connection between preeclampsia and gut microbiota, could provide potential therapeutic pathways for preventing or delaying the occurrence and development of preeclampsia. However, the human gut microbiota is quantitatively large and structurally complex, and its composition and function are impacted by diet, environmental factors, genetic factors, and so on. It is hoped that in the future, intestinal microecology will provide a new target for the prevention and treatment of maternal and infant complications related to preeclampsia.

## Author contributions

YZ: Conceptualization, Methodology, Writing – original draft. JW: Conceptualization, Methodology, Project administration, Supervision, Writing – review & editing. XW: Conceptualization, Methodology, Writing – original draft.
